# The alternative prey hypothesis revisited: Still valid for willow ptarmigan population dynamics

**DOI:** 10.1371/journal.pone.0197289

**Published:** 2018-06-06

**Authors:** Jo Inge Breisjøberget, Morten Odden, Per Wegge, Barbara Zimmermann, Harry Andreassen

**Affiliations:** 1 Faculty of Applied Ecology and Agricultural Sciences, Inland Norway University of Applied Sciences, Campus Evenstad, Koppang, Norway; 2 The Norwegian State-owned Land and Forest Enterprise, Statskog SOE, Namsos, Norway; 3 Faculty of Environmental Sciences and Natural Resource Management, Norwegian University of Life Sciences, Ås, Norway; Institut Sophia Agrobiotech, FRANCE

## Abstract

The alternative prey hypothesis predicts that the interaction between generalist predators and their main prey is a major driver of population dynamics of alternative prey species. In Fennoscandia, changes in climate and human land use are assumed to alter the dynamics of cyclic small rodents (main prey) and lead to increased densities and range expansion of an important generalist predator, the red fox *Vulpes vulpes*. In order to better understand the role of these potential changes in community structure on an alternative prey species, willow ptarmigan *Lagopus lagopus*, we analyzed nine years of population census data from SE Norway to investigate how community interactions affected their population dynamics. The ptarmigan populations showed no declining trend during the study period, and annual variations corresponded with marked periodic small rodent peaks and declines. Population growth and breeding success were highly correlated, and both demographic variables were influenced by an interaction between red fox and small rodents. Red foxes affected ptarmigan negatively only when small rodent abundance was low, which is in accordance with the alternative prey hypothesis. Our results confirm the important role of red fox predation in ptarmigan dynamics, and indicate that if small rodent cycles are disrupted, this may lead to decline in ptarmigan and other alternative prey species due to elevated predation pressure.

## Introduction

During the last decades of faunal range shifts, population declines and changes in community structures and interactions have been attributed to climate warming in Scandinavia [1]. Concern has been raised about range expansions of typical “southern species” (e.g. red fox, European brown hare- *Lepus europaeus*, European badger *Meles meles*), range contractions and decline of “northern species” (e.g. willow ptarmigan, rock ptarmigan *Lagopus muta*, mountain hare *Lepus timidus*, arctic fox *Vulpes lagopus*) and disruption of small mammal population cycles [1–6]. Particularly strong impacts of climate change are expected in mountainous habitats, partially due to an above average temperature increase in higher altitudes [7, 8] and an inevitable contraction of habitat area following an elevated altitudinal treeline. It is, however, difficult to disentangle the relative effects of climate change and other environmental stressors (e.g. changes in human land use). An example of this is the red fox, whose increase in density and range expansion in Scandinavia has been attributed to several forms of anthropogenic influence, including climate change, mesopredator release, ungulate overabundance, human land use, and altered human hunting pressure [9–12]. Irrespective of the cause, the expansion of red foxes towards higher altitudes may entail direct negative impacts on mountain wildlife communities through increased competition and predation [13, 14]. Furthermore, more complex negative effects may arise as a consequence of predator-mediated indirect interactions among prey species [15, 16], for instance, “apparent competition” may arise when two or more prey species suffer from the presence of the others via shared predators, resulting in a negative reciprocal impact on population growth. In “apparent amensalism”, one of the prey species is negatively affected by a shared predator, but not the other [17].

Further increase in mammalian generalist predators in the Scandinavian mountains are expected in the future due to rising temperatures [1], along with disturbances in the dynamics of small rodents due to altered winter conditions [18, 19]. In order to predict the potential consequences of environmental changes on mountain wildlife communities we need to enhance our current knowledge of the direct and indirect community interactions. Hence, in this paper, we analyzed nine years of population census data to investigate whether the population growth of a declining alternative prey species, the willow ptarmigan, is related to an interaction between red foxes and their main prey, small rodents.

The alternative prey hypothesis was presented as an explanation of the synchronous population cycles of small rodents and other small herbivores [20–22]. That is: generalist predators synchronize herbivore prey dynamics through prey switching during the crash phase of their main prey, with less influence on alternative prey species at high main prey densities [20]. Numerous studies support the hypothesis [23–27], and some of the strongest evidence stem from studies of mammalian generalist predators, mainly red fox and pine marten, boreal forest grouse (capercaillie and black grouse) and mountain hares. Angelstam et al. [20] found support for APH-based predictions, i.e. that red fox shifted diet when vole abundances declined, and inverse correlations between vole abundance and the mortality rates of black grouse and mountain hares. In addition, predator removal experiments conducted by Marcström et al. [28] showed that the synchrony between vole abundance and grouse breeding success vanished once red foxes and pine martens were removed. Somewhat similar patterns were observed in another experiment, where supplemental feeding of generalist predators during vole decline prevented a reduction in forest grouse chick production [25].

Regarding willow ptarmigan population dynamics, the general impact of predation and its relationship with rodent cycling has been disputed. Kausrud et al. [19] observed a strong link between the collapse of small rodent cycles during the 1990s and climate change mediated by altered snow conditions during winter. Parallel changes in the dynamics of ptarmigan and other bird communities were attributed to “shared predators being an important part of the cyclic and synchronous behavior of the system”. Later, Selås et al. [29] stated that “increased predation on eggs and chicks as the causal link between climate change and grouse density as proposed by Kausrud et al. [19] may be incorrect”. Selås et al. [29] argued that although predation may enhance cycle amplitudes in grouse, fluctuations in food plant quality is a major influential factor generating synchronized dynamics in rodents and grouse. They argued that in years following high seed production in bilberry (*Vaccinium myrtillus*)—a main food plant of ptarmigan in summer—the nutritional quality of this plant for herbivores is enhanced due to reduced chemical defenses. They inferred that warm summers during the bilberry masting year and the year before would restore the level of chemical deterrents quickly and therefore affect grouse reproductive output negatively. Partly contrasting this, a long-term populations study of boreal forest grouse documented that breeding success increased significantly along a 29-year long trend of warmer spring temperatures [30]. Still, other factors and mechanisms have been proposed as important drivers of grouse dynamics and population trends. Over a 4-year study period, Henden et al. [31] did not observe an anticipated positive response in a willow ptarmigan population during one rodent peak. Based on this, and observed effects on ptarmigan habitat occupancy patterns, Henden et al. [31] concluded that factors other than changing rodent population dynamics may be responsible for the declining trend in Scandinavian ptarmigan. Then again, Kvasnes et al. [32] observed a pronounced large-scale spatiotemporal synchrony in ptarmigan recruitment that corresponded with variation in rodent abundance. However, an effect of spring/summer climate (North Atlantic Oscillation, NAO-index, in May, June and July) had a stronger effect on recruitment of ptarmigan than rodents. Based on these results, Kvasnes et al. [32] concluded that the link between rodent and ptarmigan dynamics had been weakened following the collapse of rodent cycles during the 1990s, and that climate changes lead to desynchronization.

Interpretations and conclusions concerning the ptarmigan-rodent-predator relationship has been weakened by a lack of information of one of the main actors in the system; the predators. The fact that no studies of ptarmigan population dynamics have yet included predator abundance as a predictor variable is an obvious limitation in our knowledge of how the system works. In our study, we combined census data on ptarmigan, rodents and red fox to examine spatiotemporal patterns in ptarmigan population growth. We addressed the APH-hypothesis and evaluated its relevance for ptarmigan dynamics by testing the following prediction: that ptarmigan population growth depends on an interacting effect of abundances of rodents and mammalian generalist predators. According to APH, predation impact from generalist predators should be limited during periods of high availability of main prey, and thus, we expect that ptarmigan growth is determined by an interaction between rodents and predators.

## Material and methods

### Study area and focal species

**Fig 1:**
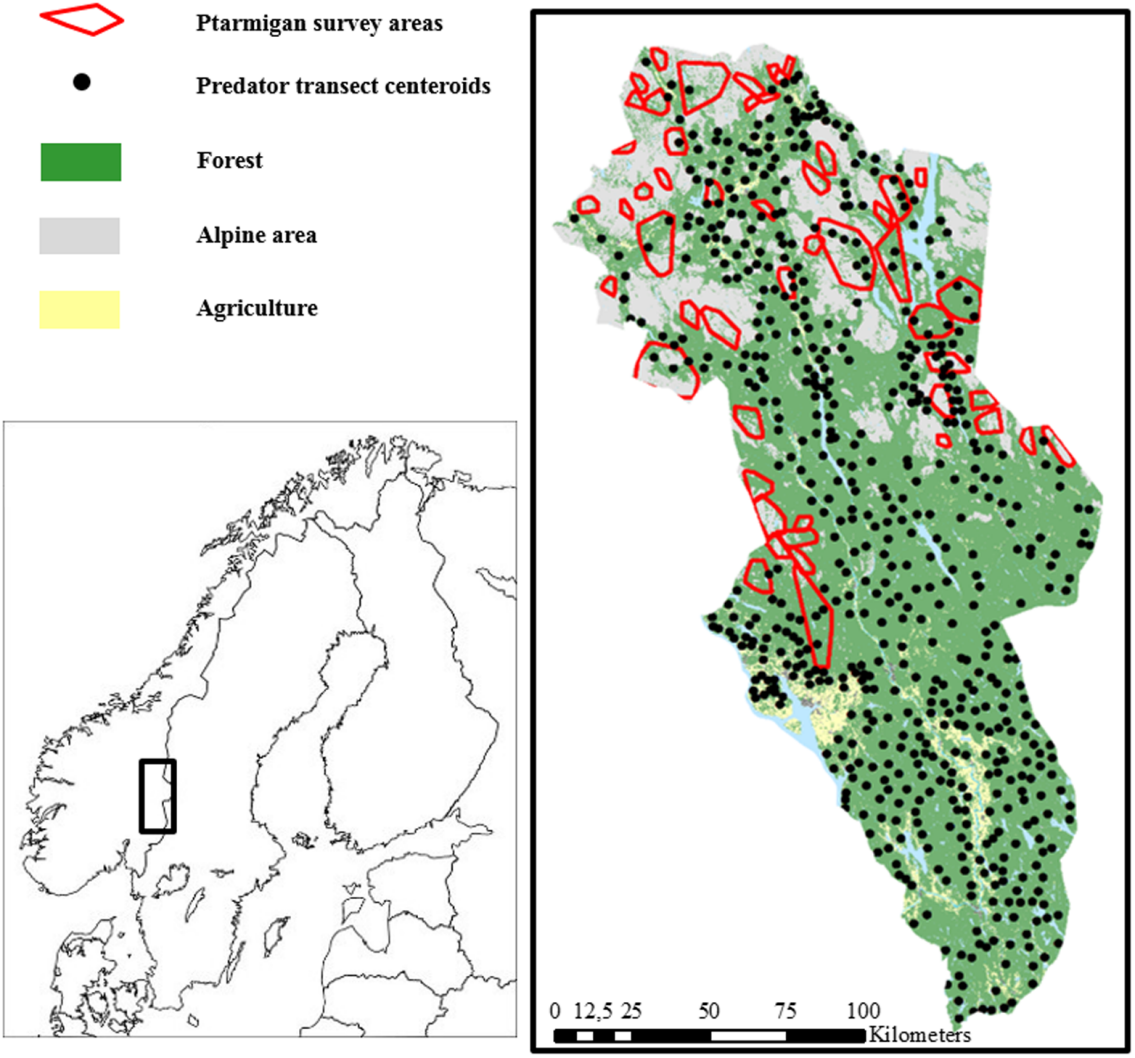
Overview of locations of ptarmigan survey areas and predator snow tracking transect centroids within the county of Hedmark, southeast Norway.

Our study was carried out in Hedmark County (27 400 km^2^, 61° N 11° E), southeastern Norway (Fig 1), in the boreal zone [33]. The southern part of the county is less mountainous and consists of a mosaic of farmland and commercially managed conifer forests, whereas fragmented alpine areas are in the northern part, covering approximately 19% [34] (Fig 1). Suitable habitat for ptarmigan at and above the altitudinal treeline is characterized by presence of willows (Salix spp.), dwarf birch (*Betula nana*) and ericaceous shrubs. The treeline is situated at elevations of approximately 800–1000 m, and the highest mountain peak (“Rondslottet”) is 2178 meters above sea level. The climate is classified as semi-continental with mean temperatures of -13 °C in January and 13 °C in July in the northern parts of the study area [35]. Potential mammalian predators present in the study area are the red fox, pine marten (*Martes martes*), stoat (*Mustela erminea*), least weasel (*Mustela nivalis*) and wolverine (*Gulo gulo*). Common avian predators are hooded crow (*Corvus cornix*), raven (*C*. *corax*), rough-legged buzzard (*Buteo lagopus*), gyrfalcon (*Falco rusticolus*) and golden eagle (*Anquila chrysaetos*). Prevailing small rodent species are Norwegian lemmings (*Lemmus lemmus*), tundra voles (*Microtus oeconomus*) and bank voles (*Myodes glareolus*).

The red fox is a medium sized and widely distributed carnivore with a broad diet including voles, hares, grouse and ungulates [36, 37]. Small rodents constitute the main food of foxes during peak years, but when rodents are scarce, foxes typically scavenge on carcasses [12, 37]. The male foxes move extensively during the mating period (January to February), and are often found far outside their own territory, probably attracted by females in oestrus, [38]. Kauhala [39] observed an average litter size of 5.1 pups, while in a scarce rodent year, Lindström [40] found a litter size of 3.4. Juvenile foxes disperse in autumn and winter from an age of ca 6 months, and male foxes move further than females [41, 42]. Foxes have in general smaller home ranges and disperse over shorter distances at high population densities than foxes living at lower densities [41–43].

Willow ptarmigan, a medium-sized grouse, is distributed in alpine tundra habitats across the northern hemisphere [44]. Males defend a breeding territory in spring [45] and within these territories 8–12 eggs are hatched by the end of June [46, 47]. Willow ptarmigan usually pair monogamously and Wittenberger [48] showed more male parental care among willow ptarmigan than cocks of any other grouse species. Brood break-up take place from late September [49].

The most pronounced biomass cycle in the boreal forest ecosystem of Scandinavia is the regular 3–4 year cycles of vole populations. Hence, voles are often considered a main driving force governing the dynamics of this system, and their population cycles may have marked effects on their predators with potentially cascading effects on other trophic levels [24]. Vole cycles are correlated with several other components of the system such as seeds, berries, specialist predators and small game (grouse and hare; see e.g. [3, 50–53]). The dynamics of small rodents in Scandinavia changed during the mid 1980ies and onwards, as fluctuations became more dampened and irregular [5]. However, the cycles seem to have reappeared in many areas during the last decade [54, 55].

### Census data

Line transect surveys of willow ptarmigan were conducted in early August 2005–2014 by the use of pointing bird dogs. During this period, an average of six annual counts (SD = 2.6) were conducted in 48 different survey areas. The size of the survey areas averaged 56.0 km^2^ (SD = 61.1), and in each area, an average of 15.4 (SD = 10.9) transect lines ($\bar{x}=3.2\;\text{km}\;\pm \;1.1\;\text{SD}$) with a total length of 47.1 km (SD = 34.5) per survey area were monitored. The dog handlers noted all observations of willow ptarmigan (i.e. group size and location), and whether or not small rodents had been observed along the transect lines.

We estimated predator track frequencies along 2.95 km (SD = 0.5) long snow tracking transect lines in January in the period 2006 to 2014 (Fig 1). The transect lines were part of a nationwide monitoring program for Eurasian lynx (*Lynx lynx*) and were based on voluntarily work from members of the Hedmark Chapter of the Norwegian Association of Hunters and Anglers. All tracks crossing the transect lines were recorded, including red fox. The transect line density was 3–4 lines per 100 km^2^ [56]. Of a total of 621 different lines, 281 to 484 lines were surveyed annually during favorable snow conditions, i.e. 2–5 days after snowfall.

### Statistical analysis

For each survey area and year, we calculated a density index for willow ptarmigan by dividing the total number of flushed birds by the total length of the surveyed transect lines (i.e. birds per km of survey). Willow ptarmigan population growth rates (r) were defined as the logarithm of the density index in year t divided by density index in year t-1 (r = ln(N_t_/N_t-1_)). As indices of breeding success, we used the number of juvenile ptarmigan among all counted birds per survey area. The surveys were conducted prior to brood break-up, and thus, single birds or pairs were most likely adults that had lost eggs or chicks. The numbers of juveniles per brood was estimated by subtracting two adults from each group of birds. For each survey area and year, we calculated small rodent density indices by dividing the number of transect lines where rodents had been observed with the total number of surveyed transect lines. Although the line transects varied in length; transects with rodent observations were on average 56 m shorter than transects with no observations, we observed no effect of this variation on the probability of detecting small rodents (*t* = 0.59, p = 0.557).

From the snow tracking transects, we calculated track frequency indices for red fox by dividing the number of tracks per km with the number of days since last snowfall. Because the predator sampling took place some distance from the ptarmigan areas, we used the inverse-distance weighting (IDW) method for spatial interpolation of predator tracks. This gave predicted index values for red fox per survey area and year. The basic assumption for this method is that the value of a non-sampled location is the average of known values within neighboring surveyed points, inversely weighted with the distances between sampled and non-sampled locations. All variables were assessed by the use of ArcGIS [57].

We analyzed the growth rate using a linear mixed effect model (LME), and breeding success using a generalized linear mixed-effect model (GLMM) from the lme4 package [58]. Willow ptarmigan growth rate and breeding success were used as dependent variables and track indices of red fox and small rodent indices as explanatory variables. All independent variables were from year t, i.e. from January for red fox and August in the same year for rodents. Hence, the survey data included in the models were collected 6–7 months apart. Despite the different timing of the surveys, we argue that the data were valid for testing APH predictions: Red fox have been shown to affect forest grouse mainly through predation of eggs and chicks, i.e. during May-June [20, 21, 28, 59, 60]. Our red fox density index data were collected prior to their reproduction period, and probably corresponded quite well with the relative distribution of predators during the following spring. If red fox had been surveyed after reproduction, the data would probably reflect spatial differences in red fox breeding success rather than relative abundances and predation impact during the most critical period for ptarmigan. Following this argument, the optimal timing of the rodent surveys would be spring rather than late summer. We did not have access to spring data, and our only alternative would be using rodent data from the preceding august survey. However, due to the very high and unpredictable winter mortality in Scandinavian small rodents [18, 61–63], we considered that survey data from August the same year served as a better proxy to spring densities. Willow ptarmigan survey area was set in the models as a random term. We used the same sets of models to analyze variation in breeding success and growth rates (Table 1). The data was analyzed using R [64]. We selected the most parsimonious model using Akaike Information Criterion (AIC) values and Akaike weights [65]. The best models had the lowest AIC—and the highest AIC weight values.

**Table 1:** Akaike information criterion (AIC), ΔAIC and AIC_w_ selection summary of four models examining the contribution of the following explanatory variables to willow ptarmigan growth rate and breeding success: Effects of rodent abundance (ROD) and red fox density index (RF).

		Growth rate			Breeding success		
Model	Explanatory variables	AIC	ΔAIC	W	AIC	ΔAIC	W
**M1**	RODxRF	586.1	0	0.97	2381.7	0	1.00
M2	ROD+RF	593.5	7.4	0.02	2393.6	11.9	0.08
M3	ROD	602.7	16.6	<0.01	2417.5	35.8	<0.01
M4	RF	626.6	40.5	<0.01	2524.9	143.2	<0.01
M0	NULL	635.5	49.4	<0.01	2559.8	178.1	<0.01

Data were collected in Hedmark county, SE Norway in 2006–2014. The most parsimonious model (M1) is marked with bold font.

## Results

**Fig 2:**
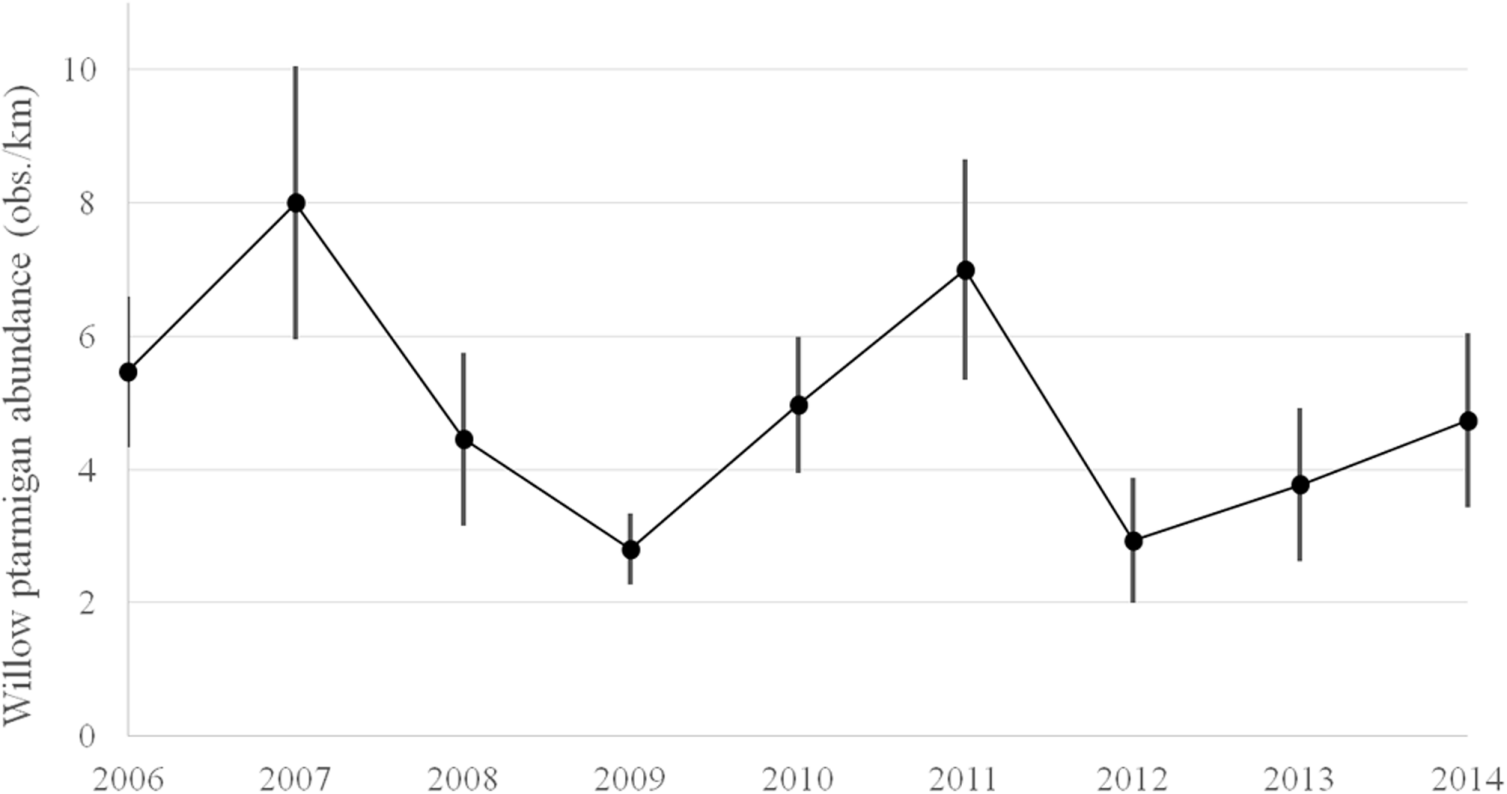
Temporal patterns of willow ptarmigan population density indices (number of birds observed per km ± 2SE error bars) obtained from 48 survey areas in Hedmark county, SE Norway.

**Fig 3:**
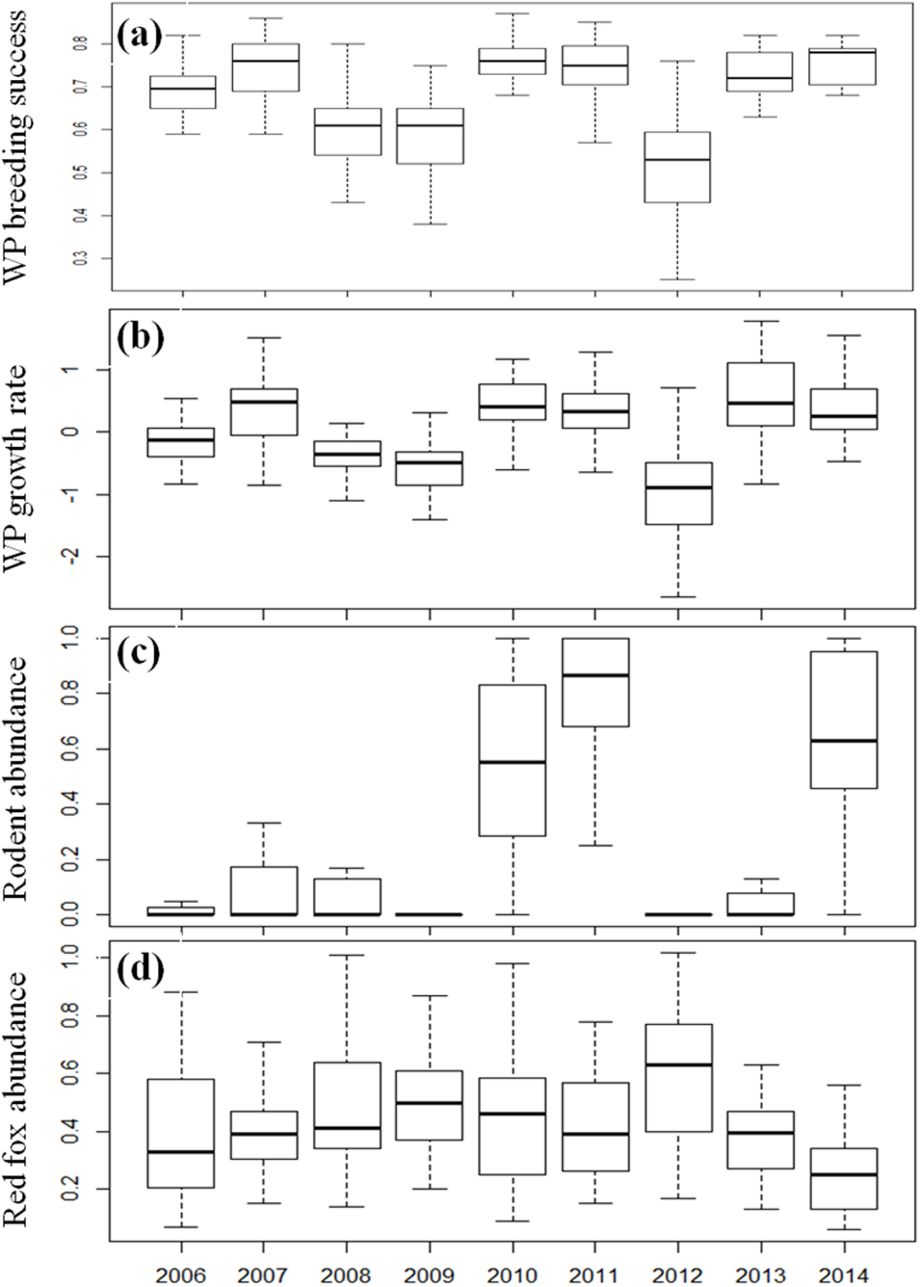
a, b, c and d. Temporal patterns of willow ptarmigan (WP) breeding success, population growth (r = ln (λ)), rodent abundance and red fox abundance indices obtained from 48 survey areas in Hedmark county, SE Norway. Each column contains the extreme of the lower whisker, the lower quartile, the median (black line), the upper quartile and the extreme of the upper whisker for each year. Breeding success was expressed as the proportion of grouse chicks, i.e. the number of chicks divided by the total number of bird observations. Rodent abundance indices were calculated for each survey area by dividing the number of ptarmigan transect lines where rodents had been observed with the total number of surveyed transect lines (i.e. max = 1.0 and min = 0.0). We used the inverse-distance weighting (IDW) method for spatial interpolation of red fox tracks based on data from 621 snow tracking transect lines that were distributed throughout the county (see Fig 1). Outliers are exluded from the box plot.

The ptarmigan population density index varied markedly between years and between areas (Fig 2). During the study period, three clear peaks occurred: in 2007 (8.0 per km, SD = 5.9), 2011 (7.0 per km, SD = 5.3) and 2014 (4.7 per km, SD = 3.2). The lowest average densities were observed in 2009 (2.8 per km, SD = 1.6) and 2012 (2.9 per km, SD = 2.7). Population growth rates were strongly and positively correlated with breeding success (R^2^ = 0.42) (Fig 3a and 3b). The rodent abundance indices (Fig 3c) exhibited high amplitudes that were synchronous with ptarmigan density: i.e. markedly low densities in 2009 (0.02, SD = 0.1) and 2012 (0.01, SD = 0.02), and peaks in 2011 (0.80, SD = 0.2) and 2014 (0.65, SD = 0.3), see (Fig 3). The red fox index varied less among years (Fig 3d).

**Fig 4:**
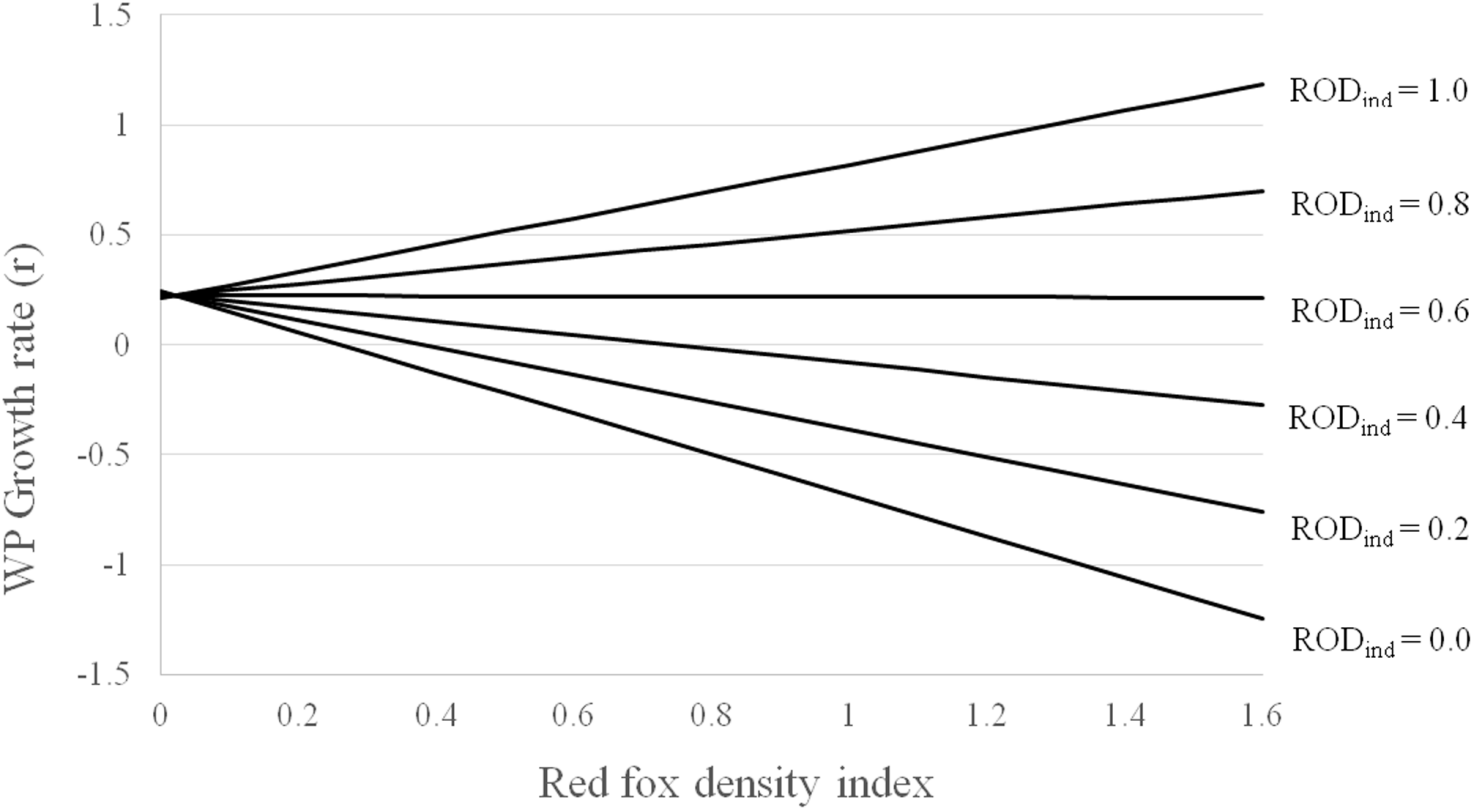
Predicted values from a model of growth rate (r) of willow ptarmigan observed in August transect counts (see Table 2). Explanatory variables were indices of red fox (tracks per km transect lines/days since last snowfall) and rodent abundance. The lines depict predicted growth rates given rodent indices (ROD_ind_) from 0–1, and red fox indices from 0–1.6.

For both ptarmigan population growth and breeding success, the best models included the interaction between rodent and red fox abundance, as expected (Tables 1 and 2). As shown in Fig 4, the negative impact of red fox on ptarmigan appeared when small rodent densities were low. During years of high rodent density, breeding success and population growth of ptarmigan were high, presumably because the impact of red fox was low. Markedly higher AIC-values were obtained from models with only one predictor variable (Table 1).

**Table 2:** Parameter estimates explaining population growth and breeding success in willow ptarmigan.

	Growth rate			Breeding success		
Predictor variable	Estimate	SE	t-value	Estimate	SE	z-value
Intercept	0.24	0.10	2.34	-0.31	0.02	-16.37
ROD	-0.03	0.25	-0.14	0.04	0.03	1.45
RF	-0.92	0.20	-4.60	-0.22	0.03	-6.28
RODxRF	1.54	0.50	3.10	0.24	0.06	3.73

Parameter estimates from the best model explaining population growth and breeding success in willow ptarmigan in Hedmark county, Norway, 2006–2014 (see Table 1).

## Discussion

In our study, population growth and breeding success of willow ptarmigan were highly correlated, and both variables were influenced by an interaction between the red fox (a generalist predator) and small rodents. This similarity emphasizes the close relationship between variation in population growth and recruitment rates. This is a typical feature of the so called high reproductive species, whose low annual survival, high fecundity and early maturity produces high population fluctuations that are typically determined by variation in recruitment [66]. Few ptarmigan fail to produce a clutch [67], and therefore recruitment rates in autumn are mainly determined by nest losses and juvenile survival [68–71]. Our results indicate that predation from mammalian generalists—especially red fox—is a significant influence in this stage, and this is in accordance with previous research on willow ptarmigan in North America [70].

In Fennoscandia, data on nest losses and chick survival in willow ptarmigan are somewhat limited, but some evidence suggests that predation is highly variable and partially caused by mammals [69, 72–75]. The role of predation on forest grouse (capercaillie and black grouse) is better known, and mammalian generalists are identified as the main threat to eggs and chicks in previous studies [20, 60, 76–79]. Furthermore, a stronger impact on recruitment rates than on adult survival of forest grouse was demonstrated in two generalist predator removal experiments in Scandinavia [28, 80]. Our study demonstrates that mammalian generalist predators may exert a comparable impact on willow ptarmigan. Still, somewhat different patterns of predation impact could be expected between prey species in alpine- and woodland habitats due to differences in carnivore community composition. Regarding predators, the most pronounced difference is the relatively lower abundances of mammals versus birds in alpine areas, and thus, mammalian predator impact on ptarmigan could be lower than for forest grouse [72, 81, 82]. However, several studies suggest that mammalian predator influence is increasing in Scandinavian mountains [9]. In particular, the red fox has received attention due to its increasingly negative impact on the arctic fox [10, 14]. The particular importance of red fox predation was demonstrated by the marked population increase in several small game species during an epizootic of sarcoptic mange that significantly reduced red fox abundance during the 1970ies and 1980ies [77, 83]. Although the red fox is a typical generalist predator, previous studies have shown a marked influence of fluctuations in main prey, voles, on their population dynamics [2, 84]. Nevertheless, the dynamics of red foxes did not seem strongly influenced by vole fluctuations in our study (Fig 3). We believe the apparent lack of correlation may be caused by a stabilizing effect of an increased availability of ungulate carrion. Ungulate densities have increased markedly in Scandinavia during the last decades, and several studies suggest this is an important resource for red foxes during periods of low prey availability [12, 37, 51, 85].

According to our analyses, red fox had a negative effect on ptarmigan growth rates and breeding success only when rodent abundances were low. This is in accordance with APH, as the predators are assumed to exert little influence on alternative prey species, e.g. ptarmigan, when the main prey densities are high. The APH predicts that a change in predation impact on alternative prey is mainly caused by a functional response of the predators. In our case, it is difficult to disentangle the relative effect of a functional or a numerical response based on the generalist predator snow tracking index, as the number of crossing tracks on transects is a product of the number of individuals present (numerical) and their individual travel distances (functional). Hence, the index may therefore better reflect the total response of the predators. Nevertheless, our analyses suggest that the functional component of the predator response plays an important role in their impact on ptarmigan, as the best models show an interaction between red fox and small rodent abundance on ptarmigan growth. The positive relationship between red fox and ptarmigan at high rodent densities, and the negative association at low rodent densities, would probably not occur unless there was a diet shift among the predators.

## Conclusions

In this paper, we have shown a clear relationship between the breeding success and growth rate of willow ptarmigan and its dependency on the interaction between the abundance of small rodents and a mammalian generalist predator; the red fox. Our results suggest that willow ptarmigan are sensitive to potential changes in the cyclic dynamics of small rodents and increase in predator abundance and that further decline in ptarmigan and other alternative prey species may occur if rodent cycles become dampened in the future. Still, although we have demonstrated linkages among a generalist predator and its main and alternative prey, more research is needed to document and quantify predation impact, the relative importance of different predator species, and the potentially interactive effects of predation and other influential factors. Furthermore, the relative influence of climate change versus other types of human influence on community dynamics in mountain ecosystems warrants further attention.

## Supporting information

S1 Data set (CSV)
